# Preparation of an Adhesive in Emulsion for Maxillofacial Prosthetic

**DOI:** 10.3390/ijms11103906

**Published:** 2010-10-13

**Authors:** Judith A. Sánchez-García, Alejandra Ortega, Federico H. Barceló-Santana, Joaquín Palacios-Alquisira

**Affiliations:** 1 Laboratorio de Materiales Dentales y Biomateriales, División de Estudios de Posgrado e Investigación (DEPeI), Facultad de Odontología, Universidad Nacional Autónoma de México (UNAM), Circuito de la Investigación Científica s/n, Delegación Coyoacán, C.P. 04510 México City, D.F., Mexico; E-Mail: barcelo@servidor.unam.mx; 2 Laboratorio 108 de Fisicoquímica Macromolecular, Departamento de Fisicoquímica, Edificio D, Facultad de Química, Universidad Nacional Autónoma de México (UNAM), Circuito de la Investigación Científica s/n, Delegación Coyoacán, C.P. 04510 México City, D.F., Mexico; E-Mails: eikkaas@hotmail.com (A.O.); polylab1@servidor.unam.mx (J.P.-A.)

**Keywords:** pressure-sensitive adhesive (PSA), maxillofacial prostheses, emulsion polymerization, acrylic monomers, drying kinetics

## Abstract

Maxillofacial prostheses is a dental medicine specialty aimed at restoring anatomical facial defects caused by cancer, trauma or congenital malformations through an artificial device, which is commonly attached to the skin with the help of an adhesive. The purpose of our research was to develop a pressure-sensitive adhesive (PSA) based on acrylic monomers, characterizing and determining its drying kinetics, that is to say the time it takes to lose 50 to 90% of its moisture. The adhesive synthesis was realized by means of emulsion polymerization; the composition of formulations was: (AA-MMA-EA) and (AA-MMA-2EHA) with different molar ratios. The formulation based on (AA-MMA-2EHA) with 50 w% of solids, presented good adhesive properties such as tack, bond strength, and short drying time. We propose this formulation as a PSA, because it offers an alternative for systemically compromised patients, by less irritation compared to organic solvent-based adhesives.

## 1. Introduction

Maxillofacial prosthetic devices play a crucial role in the rehabilitation of patients that have suffered severe facial defects [1], caused by cancer, trauma [2], disease [3,4–6] or congenital malformations [2].

Prosthetic devices can be secured with the help of adhesives, mechanical means [7], craniofacial implants and [1] anatomic tissue [8]. Adhesives are an effective and commonly used method [1,7–10]. Medical products that involve adhesion to the skin [11] or adhesives that attach to human skin [12] are known as pressure-sensitive adhesives (PSA), defined as viscoelastic materials, which in their dry state at room temperature can adhere strongly to a wide variety of substrates by application of slight pressure [11] for a short period of time [13–15] without activation by water, heat, or solvent [16].

Nowadays, PSA for skin contact applications are mostly made of acrylic polymers because they are less irritating to skin [17]. In the field of maxillofacial prosthetics in medicine and dentistry [2], the adhesives are used to fix and/or to hold the artificial part or prosthesis to the skin.

Usually, these devices are made of elastomeric material [2,6,18] such as poly dimethyl-siloxane (PDMS) [3,4–6]. The success of a facial prosthesis frequently depends on several factors including stability, support and retention; this last factor being the most important [1,8,18]. Relatively little work has been done on the development of new and improved adhesives for this purpose. We continue to use the same materials introduced decades ago, like silicone, introduced in 1950 [4] and other materials adapted with their inherent inadequacies from non medical applications [10] such as organic solvent-based adhesives for example ethyl acetate [8]. Dermatological problems such as skin sensibility and the difficulty of removing all the adhesive residues are some of the factors that affect the adhesion of material to the skin and its duration [1]. Also, the lack of a good adhesion frequently creates a visible margin between prosthesis and the skin; this phenomenon is known as debonding or peel [9]. The study of adhesion to skin is complex because of the physiology, biochemistry and histological conditions involved. The existing information about the nature, behavior and biocompatibility of pressure-sensitive adhesives for use in maxillofacial prostheses is inadequate and incomplete [10]. However, its use keeps growing despite the lack of information on available products [7,10]. The purpose of our research was to develop a pressure-sensitive adhesive (PSA) based on acrylic monomers, characterizing and determining its drying kinetics, that is the time it takes to lose 50 to 90% of its moisture.

## 2. Experimental Section

### 2.1. Materials

The monomers used for the emulsion polymerization reactions were 2-ethylhexyl acrylate (2EHA), methyl methacrylate (MMA), acrylic acid (AA), ethyl acrylate (EA). All of these were reagent grade obtained from Sigma Aldrich. The ionic surfactant used was sodium dodecyl sulphate (SDS; Aldrich). The initiator of the reaction was potassium persulfate (KPS; Monterrey). Sodium bicarbonate was used as buffer (NaHCO_3_; J.T.Baker). Hydroquinone (Barsa) was used as an inhibitor, to stop conversion in the samples. Nitrogen gas (Linde México) was used to purge the reaction mixture. Sodium hydroxide (NaOH; J.T.Baker) was used to remove the inhibitor monomers. Distilled water was used in each experimental polymerization formulation.

Water-based latex emulsion (Pros–Aide®, Factor II Inc., Lakeside, Ca, USA) was used as commercial reference and (Dragon Skin®; Smooth On) silicone were used in the drying kinetics.

Micro-porous test (CODIFARMA México) was used as control, and isopropyl alcohol (Merck México) was used to clean glass substrates and balls during the ball rolling test.

Phospho-tungstic acid (Sigma Aldrich) was used in the size of latex particles determination.

### 2.2. Preparation of the Adhesive Emulsion

The monomers MMA, EA, 2EHA were washed with a sodium hydroxide solution (5% w/v) to remove the inhibitor. Emulsion polymerization was the method used to develop the adhesive formulation [19]. Seven polymerizations were carried out using the recipes presented in Table 1. As a general procedure for all formulations; 1.3 × 10^−3^ moles of the emulsifier (SDS) was dissolved in 0.83 moles of deionized water, after adding the monomers. This pre-emulsion was stirred for 15 minutes. The rest of the emulsifier, water according to each formulation and NaHCO_3,_ were placed in a three necks round flask and stirred at 250 rpm, this blend was bubbled with nitrogen gas (N_2_) for 10 minutes to eliminate the oxygen in the system and heated to 70 °C. The initiator (KPS) was added and immediately the pre-emulsion was dosified in the flask for a period of three hours. The reaction continued for three more hours, keeping a 70 °C constant temperature to guarantee a complete monomers conversion.

### 2.3. Measurement of the Monomer Conversion

The monomer conversions were determined gravimetrically. Samples (*m*_0_) withdrawn from the reactor during the polymerization were short-stopped with a solution of 1% hydroquinone in deionized water and then the sample was dried to constant weight (*m*_1_) at 45 °C. The solid content of the system was evaluated by the following equation.

$$s=\frac{m_{1}}{m_{0}}\times 100\%$$

### 2.4. Drying Kinetic

We studied the drying kinetics to obtain the loss rate of water in the adhesive formulations, and it was compared with a commercial adhesive, Pros–Aide®, which we call PI. A glass surface was selected (Petri dish), and its weight and area were determined, *i.e.*, 23.75 cm^2^. An aliquot (1 mL) of the emulsion adhesive sample was poured on the glass Petri dish and the weight registered. Then, the spread out sample was placed in an oven at a 25 °C constant temperature to avoid direct contact with the lab environment. The registered relative humidity average (R.H.) was 48.3%. The sample was weighted at regular intervals of time until weight became constant for the moisture calculation.

The experiment was also designed in film for the formulation B3 and PI reference, under the same conditions of temperature and humidity; glass slides and strips of Dragon Skin® silicone were used with a 18.75 cm^2^ area. The silicone strips were previously polished with 240 grit sandpaper to simulate the skin surface roughness. On each preweighed sample, 5 drops of emulsion adhesive were placed, and their weight was recorded at regular intervals of time until totally dry.

For the determination of moisture content in the drying kinetics, the following equations proposed by Strumillo and Kudra were applied:

Dry Base [20,21]:

This method is based on the constancy of sample dry matter during drying. If the total sample mass *m* at time *t* is the sum of that for dry matter *m_d_* plus the water mass *m_w_*

$$m=m_{w}+m_{d}$$

and it is considered that, by definition of moisture content dry basis *X = m_w_/m_d_* then

$$m=m_{d}X+m_{d}=m_{d}(1+X)$$

The same equation applied to the initial sample, takes the form:

$$m_{0}=m_{d}(1+X_{0})$$

By dividing the equation by *m* and *m_0_* for constant dry matter content, and solving for *X*, we arrive at the calculation equation:

$$X=\frac{m}{m_{o}}(1+X_{0})-1$$

being, as follows

$$X=\frac{m_{w}}{m_{d}}$$

We integrate the area and time in seconds to the equation to determine the drying rate for each sample.

Each weighing, to determine *m,* involved some 20–30 s in a digital balance, its readability being 0.001 g.

The operating variables, used in the drying kinetic, were: constant temperature: 25 °C; relative humidity: 48.3%; content solids of 20, 21, 22, 23, 30, 40, 50% and PI as commercial reference.

One-way analysis of variance (ANOVA) was used to test for any significant difference between the mean values of the samples tested. Post-tests (Tukey method) were used to determine whether the mean value of any particular sample differed significantly from another specified sample, while considering all the data.

### 2.5. Rolling Ball Test

In this procedure [22,23], an 11-mm-diameter stainless steel ball weighing 5.6 g was rolled down on inclined track (21°, 30′) to come into contact with adhesive face. The adhesives evaluated were B3, and PI, Micro-porous test as control, at 23 °C and 50% relative humidity (R.H.).

The test was carried out on glass plates (inclined plane), with 34.5 by 5.1 cm dimensions. Before the test, the glass plates and balls were washed thoroughly with water and soap to remove any dirt that they might have, and once dried, we cleaned them with isopropyl alcohol. Immediately after, we applied approximately 38 mg of the adhesive, spreading it with a soft bristle brush, then waited for 5 and 10 minutes drying time and immediately let the ball roll. The reported results were the average of ten determinations recorded in centimeters.

The distance traveled by the ball along the track is taken as the measure of tacky.

### 2.6. Characterization of Acrylic Latex Copolymer

The characterization of B3 formulation was carried out in order to know their physicochemical characteristics.

The samples were analyzed by proton NMR (^1^H-NMR) and FTIR spectroscopy, in order to confirm the microstructure of our copolymer and the absence of monomer impurities. The (^1^H-NMR) spectra were recorded on a VARIAN 400-MR, 400 MHz using *d*-chloroform as solvent.

Fourier Transform Infrared (FTIR) measurements were conducted using a spectrometer Spectrum RXI Perkin–Elmer, where the polymer sample films were cast on the KBr crystal to obtain spectra. Spectra were recorded in the mid infrared region (4000–400 cm^−1^) at 4 cm^−1^ resolution.

Glass transition temperatures (T_g_) were determinated on an equipment Mettler Toledo (DSC) model 821^e^, taking 10 mg sample in aluminum pans, running the test at a constant heating rate of 10 °C/min. The DSC test was performed in a −150 to 110 °C temperature range.

Thermogravimetry data was carried out using a Mettler Toledo model 851^e^ TG/SDTA, with 10 mg sample in aluminum pans. The test was performed in a 25 to 400 °C temperature range and registered with a STAR 8.1 software program.

The density was obtained by means of a F. Mantey B. México to 20 °C densimeter.

### 2.7. Particle Size and Rheological Behaviour

For samples B3 and PI, we determined the particle size and rheological behavior analyzed through viscosity.

The size of latex particles was measured by transmission electron microscopy (TEM) in a JEOL 2010 instrument. We prepared a sample of diluted latexes (2 w%) and added one drop of phospho-tungstic acid 24 hours before the test. Then three drops of emulsion sample were placed on a metal grid coated with carbon to observe the polymer particles with a good delineation of edges.

The viscosity of the latex samples was measured with a MCR 301 rheometer, temperature control unit C-PTD200, CC27 measurement system and Rheoplus V3.1 (Anton Paar) software.

## 3. Results and Discussion

The results obtained for the experimental adhesive formulations A and B showed good time stability (homogeneous emulsion); however, after about two months, a separation phase was observed in formulation A, while formulation B remained stable and homogeneous throughout six months of experimentation due to the presence of 2-ethylhexyl acrylate in formulation B, Table 1.

For each emulsion polymerization step, the mean monomer conversion was 95% in four hours after completing the monomer addition. It was also found that prolonging the reaction time could not significantly raise the monomer conversion. Therefore, it is reasonable to conclude that the polymerization can be accomplished in four hours after finishing monomer addition (Data not shown).

### 3.1. Drying Kinetics

**Figure 1:**
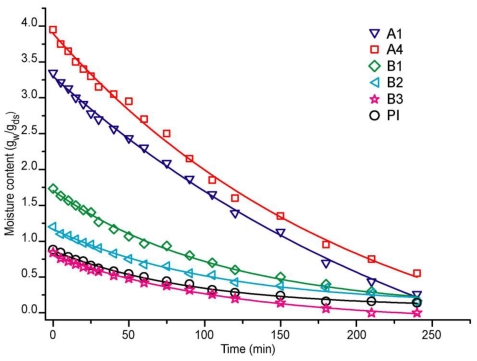
Moisture content for adhesive formulations A, B and commercial reference PI.

**Figure 2:**
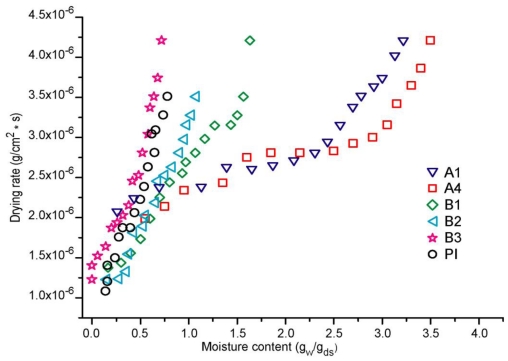
Drying rate for adhesive formulations A, B and commercial reference PI.

The drying behavior of formulations A and B were compared to that of the commercial adhesive (PI) in our experiments design. In Table 1, formulation A, we change the acrylic acid (AA) concentration to observe its effect on the kinetics performance of emulsion adhesive. We observed an increment in the water retention when the amount of acrylic acid monomer rises, since this molecule is hydrophilic and, as such, it presents a remarkable tendency to hold more water. This fact is clearly shown in Figure 1, where formulation A1 with 8 × 10^−3^ moles AA, has a lower quantity of moisture in comparison with sample A4 with (4.1 × 10^−2^ moles AA) in the formulation. Drying time was also affected, for example, A1 lost 50% of initial moisture in 120 minutes; when the amount of acrylic acid was increased, as is the case for sample A4, where less than 40% of the moisture was removed in the same amount of time under the same experimental conditions. Moreover, their calculated drying rates were different, see Figure 2: formulation A1 showed a faster capacity to lose water, showing major slope, than A4. This fact confirms that the presence of a hydrophilic monomer in A4 in the adhesive formulation delays the elimination of water, due to the capacity of poly acrylic acid (PAA) to easily form hydrogen bonds with itself and with the poly acrylates present in the formulation.

As presented in Table 1, formulation B, we decided to keep the amount of acrylic acid constant at a low (2.7 × 10^−2^ moles) level to avoid a too long drying process; besides a low concentration of acrylic acid also enhances latex colloidal stability and facilitates stronger bonds to polar substrates [15]. PAA has been shown to be a good mucoadhesive, but to achieve good adhesion, the fluidity of the formulation must be improved; this is achieved by using the monomer 2-ethylhexyl acrylate (2EHA) [24], since it produces high molar mass and tacky polymers with a low glass transition temperature. 2EHA monomer also has the hydrophobicity and fluidity required to act as a plasticizer within the adhesive formulation [15].

Figure 1 also shows the moisture behavior of formulations B. It is clear that samples with a high concentration of 2EHA (B3) take less time to eliminate their initial water content. In addition, the same test was performed on the commercial reference sample PI, showing that PI and formulation B3 with 50 w% of solids give a similar performance. 2EHA is a hydrophobic molecule; when its concentration in the adhesive formulation increases, the resulting copolymer has a major quantity of this monomer in its total composition and is more hydrophobic. Therefore, low initial moisture notably affects the drying rate, as can be seen in Figure 2, where B1 and B2 have lower drying rates compared to B3 and the reference sample PI, which presents a major slope. For formulation B3 (50 w% solids), it eliminates the solvent at a similar rate to PI, making this formulation a better candidate to develop an efficient adhesive like the reference.

In general, B formulations with (AA-MMA-2EHA) monomers behave better than A formulations, since their drying properties are similar to those of the commercial reference PI sample.

The time required to reduce moisture is an important characteristic of adhesives, because an adhesive set on facial prosthesis requires evaporation of the solvent before being set on the patient’s skin.

**Figure 3:**
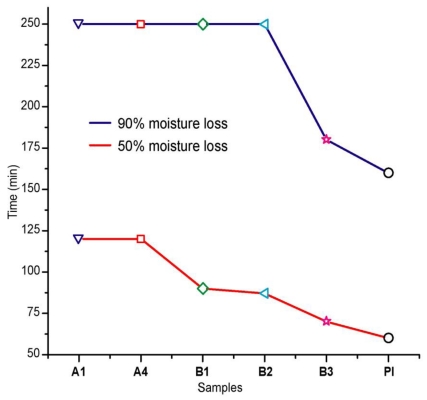
Moisture loss at 50 and 90% for adhesive formulations A, B and commercial reference PI.

In Figure 3 we present the time values corresponding to the loss of 50% and 90% of moisture. As the behavior of samples A1, A2, A3 and A4 is practically invariant at 120 minutes (50%), we only present A1 and A4 for comparison. We observed that 90% moisture loss was reached in more than 250 minutes. This may seem a long time, but it must be noted that this is an experimental probe and the thickness of our adhesive sample is larger than that used in a real prosthesis clinical test.

Samples B1 and B2 needed more time to lose moisture compared to B3 and reference sample PI. They achieved 50% moisture loss in approximately 90 minutes, while B3 needed about 70 minutes and PI 60 minutes, in our experimental setting. The same behavior was observed for the 90% moisture loss: B3 needed 180 minutes, while PI achieved it in 160 minutes *p* > 0.05. The required time for formulations A1–A4, B1 and B2 was longer than 250 minutes.

### 3.2. Drying Kinetics in Film

According to the results obtained in the previous drying kinetics, we decided to do a new test. In this test, we experimented with five drops of adhesive, for obtaining a homogeneous film. The test was performed for samples B3 and PI, with the specific purpose of establishing the drying time in a real clinical situation; that is, people who carry a maxillofacial prosthesis should be thoroughly cleaned of substrates brought into contact with the adhesive, (skin-prosthesis); later, the prosthesis should be positioned with the adhesive spread evenly by means of a soft bristle brush; after waiting for the evaporation of solvent, a second and third layer is applied following the same instructions.

**Figure 4:**
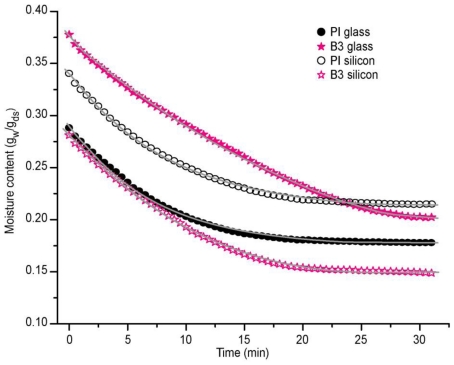
Moisture content for adhesive formulations B3 and commercial reference PI.

Figure 4 shows the moisture *versus* time graph for samples B3 and PI. Samples were prepared on glass slides and silicon. We see that the moisture loss of B3 with respect to PI on the glass slide, was a few minutes longer; PI reached a total drying time in just 18 minutes, while B3 needed about 28 minutes; *i.e.*, there was a difference of almost 10 minutes between the two samples, so we can infer that the water in the emulsion B3 showed higher interaction with the glass which led to a prolonged time of evaporation of the solvent water. However, this was not the case for the silicon sample, both samples showed similar behavior; B3 and PI took 20 minutes for total drying. Here we can infer that due to the hydrophobicity of the silicon the substrate dried more rapidly, *i.e.*, the water molecules were more exposed to the outside which facilitated their loss.

**Figure 5:**
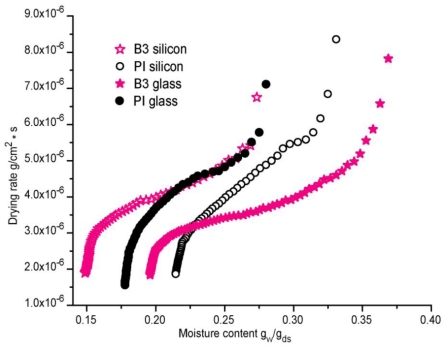
Drying rate for adhesive formulations B3 and commercial reference PI.

The drying rate graph is presented in Figure 5, where the rate of solvent loss for samples B3, in glass and silicon is very similar, with a tendency to the formation of an S shape curve and a bit slower, while the drying rate of PI is very fast, almost linear.

**Figure 6:**
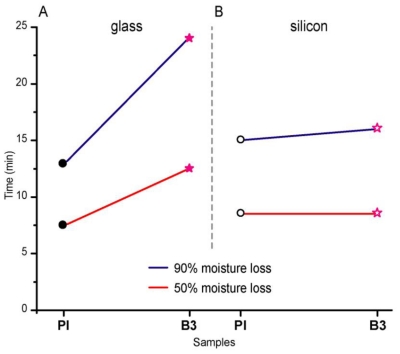
Moisture loss at 50 and 90% for adhesive formulations B3 and commercial reference PI.

Figure 6 shows the results of the moisture time loss at 50 and 90%; note that for B3, the time required to evaporate the water from glass slide was 12.5 and 24 minutes respectively, while PI required a shorter time, 7.5 and 13 minutes respectively. However, for silicon the results were different; B3 reached only a loss of 50% moisture in 8.5 minutes and 90% in 16 minutes likewise, 8.5 and 15 minutes respectively for PI.

The silicon results seem very promising as they show shorter drying times compared with glass, as they reflect the real situation of the substrate, where the adhesive should be placed.

### 3.3. Rolling Ball Test

According to obtained results of film drying kinetics, we decided to test the adhesive performance (tacky) for adhesive formulation B3, by means of the rolling ball test (see Section 2).

In clinical practice, the drying time is directly proportional to tacky, *i.e.*, after placing the adhesive on the prosthesis, it is important to wait until the solvent has evaporated which is achieved within only 5 to 10 minutes (see Figure 6) for a moisture loss of 50 and 90% respectively. During the rolling ball test at 5 and 10 minutes, the commercial reference PI showed higher adhesiveness than B3 formulation and Micro-porus. The formulation B3 showed higher adhesiveness at 5 minutes than at 10 minutes.

Therefore, we propose that the prosthesis be placed with adhesive on the skin during the first 5 minutes, when it has higher efficiency (tacky) *p* < 0.05 (see Table 2).

Due to previously reported results, we decided to characterize the B3 formulation, for their adhesive behavior and less drying time.

### 3.4. Characterization of Acrylic Latex B3 Copolymer

**Figure 7:**
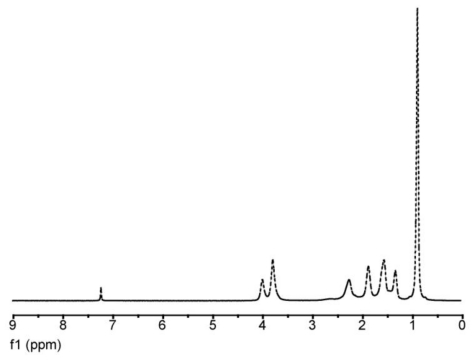
Representative ^1^H-NMR spectral scan for B3 copolymer.

The ^1^H-NMR spectral scans for the polymer, showed no peaks due to unsaturated protons between 5 and 6 ppm, which indicates the absence of residual monomer impurities. The ^1^H-NMR spectra scan of sample B3, Figure 7, showed peaks at: 0.91 ppm, 4 methyl groups, 1.31 ppm for –CH_2_– methylene group in the backbone chain, 1.60, ppm for methine groups present in the acrylic copolymer a doublet centered 3.8 ppm due to –CH_2_– methylene group in the pendant chain.

**Figure 8:**
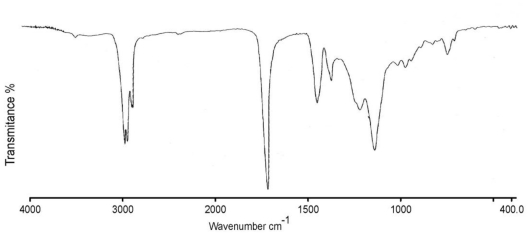
Representative FTIR spectrum of B3 copolymer.

**Scheme 1:**
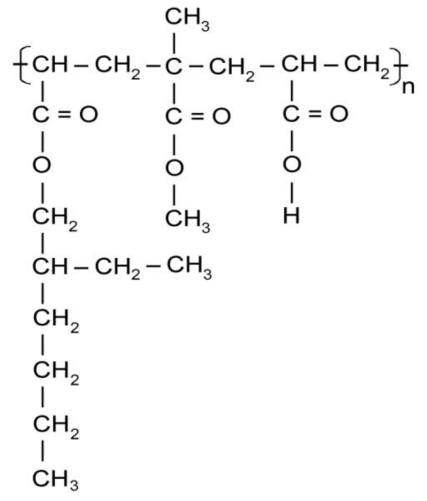
Chemical structure of B3 copolymer.

Scheme 1 shows the chemical copolymer structure in formulation B3; and its FTIR spectra confirms the chemical structure (AA-MMA-2EHA). Figure 8 shows no absorption in the characteristic C=C bond region at 1628 cm^−1^ which further indicates the absence of monomer impurities.

A short and weak peak around 3440 cm^−1^ confirms a low concentration of hydroxyl groups due to acrylic acid. We observe the presence of the compound obtained with CH_3_ characteristic peaks in 2957, 1459 and 1382 cm^−1^; characteristic peaks of CH_2_, in 2929, 2859 cm^−1^ and CH peaks in 2855 cm^−1^; the presence of COO^−^ group in 1734 cm^−1^ confirms the structure of an acrylic ester.

**Figure 9:**
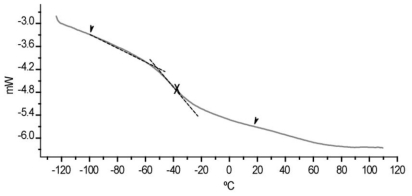
DSC (*T_g_*) data of acrylic latex film of B3 copolymer.

Figure 9 shows the DSC thermogram. The glass transition temperature *T_g_* was observed at −38.56 °C between the corresponding homopolymers and decreased with increasing monomer 2EHA concentration. However, the values of *T_g_* connects with the flexibility of the latex film. When *T_g_* increases, the film flexibility decreases.

**Figure 10:**
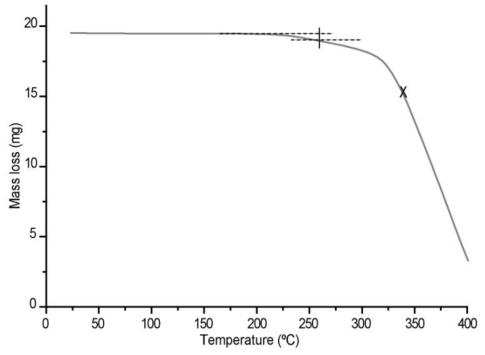
Thermogravimetry data of acrylic latex film of B3 copolymer.

From the information obtained in the thermogravimetric TG test, Figure 10 shows that there is a first loss of mass of approximately 5 w% at a 242 °C temperature and a second loss of mass due to degradation in the 320–340 °C temperature range.

The density found was 1.02 g/cm^3^.

### 3.5. Particle Size of Latex Samples

**Figure 11:**
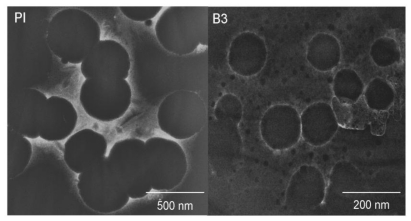
Images of latex particles by TEM for PI and B3 copolymer.

The TEM images of latex particles of samples B3 and PI show well formed spherical particles, see Figure 11. These spheres have a particle diameter (Dp) of between 100–180 nm for sample B3 and in the range of 250–400 nm for PI. The particle size distribution range of PI was calculated as the difference between the high and low particle diameter values recorded ΔDp = 150 nm, is 1.8 times higher than the particle size range observed for latex B3, ΔDp = 80 nm.

### 3.6. Rheological Behavior of Latex Samples

**Figure 12:**
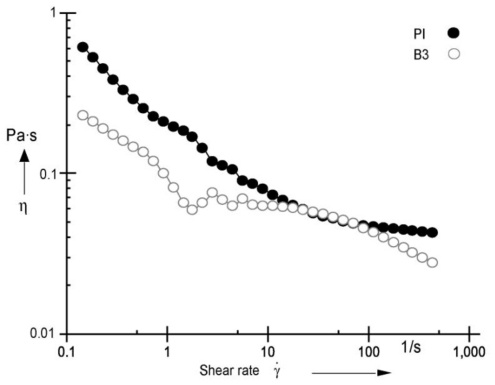
Viscosity of latex for PI and B3 copolymer.

The non-Newtonian behavior of our polymer emulsion, sample B3 and commercial reference PI, is apparent in Figure 12, whereby the viscosity, in Pa•s, decreases rapidly as the rate of shear in s^−1^, increases as expected. Polymeric emulsions become Newtonian in conduct at high shear rates. In other words, at high shear rates, the viscosity becomes or remains almost constant. At a constant 22 °C temperature, a log–log, viscosity *versus* shear rate plot, B3 sample shows an important decrement in viscosity when the shear rate γ̇ varies from 0.1 to 1.0 s^−1^, showing a change in the slope at 1.8 s^−1^. After that point increments in γ̇, did not modify the viscosity and an almost constant value of η = 0.07 Pa•s was recorded. Then at a shear rate of around 20 s^−1^, increments in the shear rate originated very small changes in viscosity. When γ̇ = 100 s^−1^, the viscosity value is as low as 0.055 Pa•s.

## 4. Conclusions

In our work we obtained a PSA; formulation B3 contains (AA-MMA-2EHA) with 50 w% of solids with good adhesiveness, tackiness and drying behavior similar to the commercial reference (PI).

We identified the control variables to modify the behavior of our adhesive: 2EHA concentration influences adhesion [24], and changes the drying rate, since the required time to eliminate the water in the formulation diminishes when the quantity of 2EHA increases. Another important factor is the amount of hydrophilic monomer present in the formulation, so if the level of acrylic acid in the formulation is high, water elimination is slower, due to formation of hydrogen bonds.

The present study offers a proposal of acrylic polymers for medical application, as an adhesive to adhere to the skin maxillofacial prosthesis. Our research shows that formulation B3, synthesized by water-based emulsion presents a good alternative for patients that have suffered severe facial damage by reduction of irritation compared with organic solvent-based adhesives.

One unrestrictedly positive aspect is that solvent-free acrylate adhesives are not only in harmony with the increasing concerns for the environment, but they even make a decisive contribution to reducing the strain on the environment because of their total omission or almost one hundred percent re-use of solvents, thereby contributing to foment the green chemistry.

## Figures and Tables

**Table 1:** Adhesive formulations synthesized by an emulsion polymerization.

Chemical reagents	Formulation A^a^				Formulation B^a^		

	A1	A2	A3	A4	B1	B2	B3
**AA**	8 × 10^−3^	1.3 × 10^−2^	2.7 × 10^−2^	4.1 × 10^−2^	2.7 × 10^−2^	2.7 × 10^−2^	2.7 × 10^−2^
**MMA**	9.5 × 10^−2^	9.5 × 10^−2^	9.5 × 10^−2^	9.5 × 10^−2^	1.34 × 10^−1^	1.34 × 10^−1^	1.34 × 10^−1^
**EA**	1 × 10^−1^	1 × 10^−1^	1 × 10^−1^	1 × 10^−1^	0	0	0
**2-EHA**	0	0	0	0	8.13 × 10^−2^	1.35 × 10^−1^	1.89 × 10^−1^
**SDS**	2.6 × 10^−3^	2.6 × 10^−3^	2.6 × 10^−3^	2.6 × 10^−3^	2.6 × 10^−3^	2.6 × 10^−3^	2.6 × 10^−3^
**KPS**	1.0 × 10^−3^	1.0 × 10^−3^	1.0 × 10^−3^	1.0 × 10^−3^	1.0 × 10^−3^	1.0 × 10^−3^	1.0 × 10^−3^
**NaHCO3**	2.4 × 10^−3^	2.4 × 10^−3^	2.4 × 10^−3^	2.4 × 10^−3^	2.4 × 10^−3^	2.4 × 10^−3^	2.4 × 10^−3^
**H2O**	4.4	4.4	4.4	4.4	3.8	3.3	2.7
**Solids content (w%)**	20	21	22	23	30	40	50

All reactions were performed at 70 °C for 6 hours.

a The amounts are in moles.

**Table 2:** Tack rolling ball test values of the B3 formulation, PI and Micro-porous samples at 5 and 10 minutes.

Samples	Drying time (min)	n	Mean distance (cm)	SD	SE
**B3**	5	10	2.76	0.52747	0.1668
**B3**	10	10	18.35714	3.31303	1.25221
**PI**	5	10	0.795	0.26714	0.08448
**PI**	10	10	0.615	0.09443	0.02986
**Micro-porus**	__	10	1.96	0.36576	0.11566

## References

[1] Kiat-amnuay S Gentelman L Khan Z Goldsmith LJ Effect of adhesive retention of maxillofacial prostheses. Part 1: Skin dressings and solvent removers J. Prosthet. Dent 2000 84 335 340 11005907 10.1067/mpr.2000.109507

[2] Kiat-amnuay S Gentelman L Khan Z Goldsmith LJ Effect of adhesive retention of maxillofacial prostheses. Part 2: Time and reapplication effects J. Prosthet. Dent 2000 85 438 441 11357068 10.1067/mpr.2001.115889

[3] Aziz T Waters M Jagger R Analysis of the properties of silicone rubber maxillofacial prosthetic materials J. Dent 2003 31 67 74 12615022 10.1016/s0300-5712(02)00084-2

[4] Bellamy K Limbert G Waters MG Middleton J An elastomeric material for facial prostheses: Synthesis, experimental and numerical testing aspects Biomaterials 2003 24 5061 5066 14559020 10.1016/s0142-9612(03)00412-5

[5] Eleni PN Krokida MK Frangou MJ Polyzois GL Maroulis ZB Marinos-Kouris D Structural damages of maxillofacial biopolymers under solar aging J. Mater. Sci. Mater. Med 2007 18 1675 1681 17483904 10.1007/s10856-007-3027-4

[6] Lai JH Wang LL Ko CC De Long RL Hodges JS New organosilicon maxillofacial prosthetic materials Dent. Mater 2002 18 281 286 11823022 10.1016/s0109-5641(01)00050-1

[7] Wolfaardt JF Tam V Faulkner MG Prasad N Mechanical behavior of three maxillofacial prosthetic adhesive systems: A pilot project J. Prosthet. Dent 1992 68 943 949 1494125 10.1016/0022-3913(92)90556-p

[8] Dahl JE Polyzois GL Irritation test of tissue adhesives for facial prostheses J. Prosthet. Dent 2000 84 453 457 11044854 10.1067/mpr.2000.109506

[9] Kiat-amnuay S Gentelman L Goldsmith LJ Effect of multi-adhesive layering on retention of extraoral maxillofacial silicone prostheses *in vivo* J. Prosthet. Dent 2004 92 294 298 15343167 10.1016/j.prosdent.2004.06.007

[10] Tam V Faulkner G Wolfaardt JF Apparatus for the mechanical testing of maxillofacial prosthetic adhesives J. Prosthet. Dent 1992 67 230 235 1538333 10.1016/0022-3913(92)90460-r

[11] Karwoski AC Plaut RH Experiments on peeling adhesive tapes from human forearms Skin Res. Technol 2004 10 271 277 15536659 10.1111/j.1600-0846.2004.00082.x

[12] Venkatraman S Gale R Skin adhesives and skin adhesion: 1. Transdermal drug delivery systems Biomaterials 1998 19 1119 1136 9720896 10.1016/s0142-9612(98)00020-9

[13] Tobing SD Klein A Molecular parameters and their relation to the adhesive performance of acrylic pressure-sensitive adhesives J. Appl. Polym. Sci 2001 79 2230 2244

[14] Creton C Pressure-sensitive adhesives: An introductory course MRS Bull 2003 28 434 439

[15] Foster A Lovell PA Rabjohns M Control of adhesive properties through structured particle design of water–borne pressure–sensitive adhesives Polymer 2009 50 1654 1670

[16] Lei CH Ouzineb K Dupont O Keddie JL Probing particle structure in waterborne pressure-sensitive adhesives with atomic force microscopy J. Colloid. Interface Sci 2007 307 56 63 17174966 10.1016/j.jcis.2006.11.036

[17] Kenney JF Haddock TH Sun RL Parreira HC Medical–grade acrylic adhesives for skin contact J. Appl. Polym. Sci 1992 45 355 361

[18] Kiat-amnuay S Waters PJ Roberts D Gettleman L Adhesive retention of silicone and chlorinated polyethylene for maxillofacial prostheses J. Prosthet. Dent 2008 99 483 488 18514671 10.1016/S0022-3913(08)60113-4

[19] Wang X Lai G Jiang Z Zhang Y Synthesis of water-soluble hyperbranched polymer and its application in acrylic latex Eur. Polym. J 2006 42 286 291

[20] Miranda M Maureira H Rodriguez K Vega-Galvez A Influence of temperature on the drying kinetics, physicochemical properties, and antioxidant capacity of Aloe Vera (Aloe Barbadensis Miller) gel J. Food Eng 2009 91 297 304

[21] Márquez CA De Michelis A Giner SA Drying kinetics of rose hip fruits (Rosa eglanteria L.) J. Food Eng 2006 77 566 574

[22] ASTM D 3121-94, Standard Test Method for Tack of Pressure-Sensitive Adhesives by Rolling Ball American Society for Testing and Materials West Conshohocken, PA, USA 1999

[23] Minghetti P Cilurzo F Montanari L Evaluation of adhesive properties of patches based on acrylic matrices Drug Dev. Ind. Pharm 1999 25 1 6 10028412 10.1081/ddc-100102135

[24] Shojaei AH Paulson J Honary S Evaluation of poly (acrylic acid-co-ethylhexyl acrylate) films for mucoadhesive transbuccal drug delivery: Factors affecting the force of mucoadhesion J. Control. Release 2000 67 223 232 10825556 10.1016/s0168-3659(00)00216-9

